# Association between Antibiotic Exposure and Type 2 Diabetes Mellitus in Middle-Aged and Older Adults

**DOI:** 10.3390/nu15051290

**Published:** 2023-03-05

**Authors:** Lei Chu, Deqi Su, Hexing Wang, Dilihumaer Aili, Bahegu Yimingniyazi, Qingwu Jiang, Jianghong Dai

**Affiliations:** 1 School of Public Health, Xinjiang Medical University, 567 Shangde North Road, Urumqi 830000, China; 2 Key Laboratory of Public Health Safety of Ministry of Education, School of Public Health, Fudan University, Shanghai 200032, China

**Keywords:** antibiotic, type 2 diabetes mellitus, hazard quotient, hazard index

## Abstract

Background: Although previous studies have shown an association between clinically used antibiotics and type 2 diabetes, the relationship between antibiotic exposure from food and drinking water and type 2 diabetes in middle-aged and older adults is unclear. ObjectivE: This study was aimed at exploring the relationship between antibiotic exposures from different sources and type 2 diabetes in middle-aged and older people, through urinary antibiotic biomonitoring. MethodS: A total of 525 adults who were 45–75 years of age were recruited from Xinjiang in 2019. The total urinary concentrations of 18 antibiotics in five classes (tetracyclines, fluoroquinolones, macrolides, sulfonamides and chloramphenicol) commonly used in daily life were measured via isotope dilution ultraperformance liquid chromatography coupled with high-resolution quadrupole time-of-flight mass spectrometry. The antibiotics included four human antibiotics, four veterinary antibiotics and ten preferred veterinary antibiotics. The hazard quotient (HQ) of each antibiotic and the hazard index (HI) based on the mode of antibiotic use and effect endpoint classification were also calculated. Type 2 diabetes was defined on the basis of international levels. Results: The overall detection rate of the 18 antibiotics in middle-aged and older adults was 51.0%. The concentration, daily exposure dose, HQ, and HI were relatively high in participants with type 2 diabetes. After model adjustment for covariates, participants with HI > 1 for microbial effects (*OR* = 3.442, 95%*CI*: 1.423–8.327), HI > 1 for preferred veterinary antibiotic use (*OR* = 3.348, 95%*CI*: 1.386–8.083), HQ > 1 for norfloxacin (*OR* = 10.511, 96%*CI*: 1.571–70.344) and HQ > 1 for ciprofloxacin (*OR* = 6.565, 95%*CI*: 1.676–25.715) had a higher risk of developing type 2 diabetes mellitus. Conclusions: Certain antibiotic exposures, mainly those from sources associated with food and drinking water, generate health risks and are associated with type 2 diabetes in middle-aged and older adults. Because of this study’s cross-sectional design, additional prospective studies and experimental studies are needed to validate these findings.

## 1. Introduction

Antibiotics have been widely used for the treatment of bacterial infections in humans and animals and for the growth of animals since Alexander Fleming discovered that penicillin can be used to treat bacterial infections in 1928. Aureomycin was subsequently found to promote growth in animals in the 1940s [1,2,3]. The overuse of antibiotics in humans and particularly in animals, in which they are used in large quantities, has led to the presence of antibiotic residues in animals. A substantial proportion of antibiotics (30–90%) is excreted in an unchanged form or as active metabolites through urine or feces in animals [4]. Our previous studies have shown that antibiotic residues in animals persist in processed foods (meat foods, livestock and poultry products, aquatic products, milk, etc.) and enter the human diet [5]. Moreover, antibiotic residues in excreted urine and feces, known as metabolite contaminants, can enter the environment (in surface water and sediment) and then human drinking water [6]. Existing studies have detected antibiotic residues in food and water environments [7].

The relationship between antibiotic exposure and type 2 diabetes mellitus (T2DM) has received increasing attention, given the adverse effects of excessive antibiotic use in humans and animals, and the similar effects of antibiotic exposure and use in middle-aged and older adults [8,9]. Some epidemiological evidence suggests that T2DM is associated with alterations in microbiota composition and function [10,11]. Specific antibiotics have been associated with perturbed glucose homeostasis in patients with T2DM [12]. However, these studies have examined the relationships between clinically administered antibiotics and T2DM, whereas the relationship between daily exposure to antibiotics from one’s diet or drinking water and T2DM remains unclear [13,14].

Many indicators are available for assessing antibiotic exposure, such as the concentration, detection rate, daily exposure dose (DED), hazard quotient (HQ) and hazard index (HI). The HI is an index for the quantitative and systematic evaluation of antibiotic exposure levels, which can provide a comprehensive indication of antibiotic exposure levels [6]. This study was conducted to assess the relationship between antibiotic exposure and the risk of T2DM in middle-aged and older people in Xinjiang, by testing urinary antibiotic exposure and then calculating the risk index through a biomonitoring method. Notably, unlike previous clinical studies based solely on questionnaire surveys, this study did not overlook the endogenous antibiotic exposure pathway.

## 2. Materials and Methods

### 2.1. Study Population

Participants were from the National Key Research and Development Program “Xinjiang Multi-Ethnic Natural Population Cohort Construction and Health Follow-Up Study,” which has been conducted by our group since 2018 [15]. In the 2019 baseline survey, subsamples of participants were recruited randomly from three townships in Huocheng County in the Yili region (Langgan Township, Sarbulak Township and Luchaogou Township) according to the main local ethnic groups. The inclusion criteria were as follows: middle-aged and older people who were 45–75 years of age; residents who had lived in the areas for more than 3 years; and those who had a non-acute onset state. The exclusion criteria were severe liver or kidney disease, or mental illness. A total of 659 middle-aged or older adults were enrolled in this study; 134 had incomplete questionnaires or lacked urine or blood glucose information. Thus, 525 middle-aged or older people were included in this study, including 145 Han, 136 Hui, 138 Uyghur and 106 Kazakh individuals. These comprised 345 middle-aged people and 180 older people, of whom 264 were men and 261 were women. All participants signed an informed consent form, and the study was approved by the Ethics Committee of the Xinjiang Uygur Autonomous Region Hospital of Traditional Chinese Medicine (2018XE0108).

### 2.2. Urine Testing and Medical Information Collection

For the field investigation, the study participants were instructed to fast before the physical examination. An amount of 12 mL of morning urine was collected from the participants, and the urine samples were stored in the dark and frozen in a −40 °C refrigerator on site after collection. Urine samples (1 mL) were purified with an Oasis HLB 96-well solid phase extraction plate and analyzed via ultraperformance liquid chromatography coupled with high-resolution quadrupole time-of-flight mass spectrometry with an isotopic internal standard in 1 mL of urine, hydrolyzed by β-glucuronidase. All antibiotics were analyzed with an HSST3 chromatography column for separation. The three phenols were separated with acetonitrile and an aqueous mobile phase in the negative ionization mode, and the other antibiotics were separated with methanol and an aqueous mobile phase with 0.1% formic acid in the positive ionization mode. Each batch was based on 96-well solid phase extraction plates. A total of 96 samples were analyzed: 92 real urine samples, 2 solvent blank samples and 2 spiked urine samples of 10 ng/mL. The solvent blank samples and the urine samples spiked with the standard were analyzed together with the real urine samples. Solvent blanks were used to monitor background interference, and spiked urine samples were used to monitor precision and accuracy. The limits of detection and limits of quantification were defined as signal-to-noise ratios of 3 and 10, respectively. The limits of detection and limits of quantification for all antibiotics ranged from 0.04 to 1.31 ng/mL and from 0.13 to 4.37 ng/mL, respectively.

Physical examinations were performed by trained nurses or physicians. A 20 mL blood sample was collected for each participant with a vacuum blood collection device with an intravenous anticoagulant. Biochemical and routine blood tests were performed with 4 mL blood samples. These tests were performed at the township health center nearest to the survey site. Whole blood samples (3 mL) were transferred to three cryotubes immediately after blood sample collection. The blood samples used to separate plasma and leukocytes were centrifuged (4 °C, 3000 rpm, 10 min) within 2 h after blood sample collection.

The questionnaires were administered by medical students who had received professional training. The baseline questionnaire was mainly the China Kadoorie Biobank study baseline questionnaire, with minor modifications based on the comments of experts from Northwestern Medical College, China [16]. The questionnaire collected information on sociodemographic information; tea and coffee consumption; alcohol intake; smoking status; dietary status (the consumption frequency of pork, beef, mutton, fried food, vegetables and fruits was referred to in the food frequency method questionnaire) [17]; passive smoking and indoor air pollution; personal and family medical history; physical activity; mental health; and female reproductive history.

### 2.3. Antibiotic Health Risk Assessment

The daily exposure dose (DED) is an indicator used to assess the daily antibiotic exposure of an organism according to the following formula for the DED of an environmental contaminant [18,19]:

$\mathrm{DED}=\frac{\mathrm{CsV}}{\mathrm{MbP}}$ (in μg/kg/day), where Cs is the antibiotic concentration in μg/L; V is the daily urine volume in L/day (daily urine volume was set at 1.70 L/day and 1.60 L/day for men and women, respectively); Mb is the body weight in kg; and P is the proportion of antibiotic excreted in urine, from the human pharmacokinetic data in an unchanged form and in a glucuronide bound form.

The acceptable daily intake (ADI) is an indicator of exposure applicable to long-term low doses of antibiotics, rather than the short-term high doses used in clinical settings. The current common standard for the ADI, established by the World Health Organization and the Food and Agriculture Organization of the United Nations in 1957, aims to establish a safe limit value to minimize the hazards threatening population health, which in turn is used to assess the risk of exposure [20]. We quantified the ADI of antibiotics by referring to the international ADI standard. With the exception of the three sulfonamides, the ADIs were established on the basis of the microbiological effects of common bacteria in the human gut microbiota. The ADIs for the three sulfonamides were established on the basis of toxicological effects.

The HQ is used to assess the health risk of each antibiotic and is defined as the ratio of daily exposure, i.e., the ratio of DED to the daily available dose ADI [21]: HQ = $\frac{\mathrm{DED}}{\mathrm{ADI}}$; HI = ∑HQ.

The HI is used to assess the cumulative health risk of combined antibiotic exposures and is based on a dose-additive concept of similar effects induced by multiple antibiotics, defined as the sum of HQs with similar effect endpoints. It is a new index for the quantitative and systematic evaluation of antibiotic exposure levels that provides a comprehensive indication of the level of antibiotic exposure. The HI is currently applied in evaluating the health risk of the long-term low-dose cumulative intake of antibiotics in humans. Because adults can be exposed to multiple antibiotics simultaneously, the HI was determined for 3 sulfonamides according to toxicological effects, 11 antibiotics according to microbial effects, 4 antibiotics for veterinary use and 10 antibiotics for preferred veterinary use, on the basis of different effect endpoints. An HQ or HI ≥1 indicates the presence of a potential health risk.

### 2.4. Statistical Analysis

The 18 antibiotics were grouped according to their antimicrobial mechanism or use, and new variables were generated via the summation of antibiotic mass concentrations in urine. The three new variables were human antibiotics (HAs), veterinary antibiotics (VAs) and preferred veterinary antibiotics (PVAs). The five new variables for antimicrobial mechanisms were tetracyclines, fluoroquinolones, macrolides, sulfonamides and phenicols. The same categories of HI were summed to generate new variables, which were divided into HI for microbiological effects, HI for toxicological effects, HI for veterinary use and HI for preferred veterinary use. Descriptive analysis provided the frequency of antibiotic testing or percentage of selected concentrations (P95, P99). The rank-sum test was used to analyze differences in blood glucose or antibiotic concentrations and other demographic variables, such as sex, age, education, income level, physical activity, diet, smoking and alcohol consumption.

The HI was classified into two groups according to hazard to human health: the HI ≤ 1 group and the HI > 1 group. Participants with fasting blood glucose values ≥7.0 mmol/L and self-reported T2DM were classified as the T2DM group, and those with fasting blood glucose values <7.0 mmol/L were classified as the non-T2DM group [22]. Variables were included in Model A as covariates if the *p*-value for the demographic variable of the total glucose or total antibiotic concentration derived from the *t*-test, ANOVA or rank test (Kruskal–Wallis test) was <0.05. Model A included the following covariates: age and income level. If the *p* value of the demographic variables of the total blood glucose or total antibiotic concentration obtained from the *t*-test, ANOVA or rank test (Kruskal–Wallis test) was <0.2, these variables were included in Model B as covariates. Model B included the variables from Model A as covariates plus sex, education, frequency of eating pork, frequency of eating mutton, frequency of eating fried food, vegetable consumption and smoking. The covariates associated with the relationship between antibiotic use and T2DM that had been studied in previous clinical settings were included in Model C. Model C included the variables in Model B as covariates plus physical activity, frequency of eating beef, fruit consumption and drinking (i.e., the covariates adjusted for in Model C were the risk factors for type 2 diabetes and all the demographic characteristics we focused on). Finally, the three models were statistically analyzed with binary logistic regression models to assess the relationship between HI and T2DM. All statistical analyses were performed in the statistical software SPSS 26. *p*-values <0.05 were considered statistically significant.

## 3. Results

All 18 antibiotics were detected in urine, with an overall detection rate of 51.0% and detection rates of individual antibiotics ranging from 0.2% to 20.0%. Urinary antibiotic concentrations ranged from below the limit of detection to above 1000 ng/mL (Table 1). Two or more antibiotics were detected in urine in 20.9% of individuals. Almost all non-T2DM populations had lower concentrations of antibiotics at the 95th percentile and 99th percentile than did the T2DM populations, except for tetracycline, norfloxacin, ofloxacin, thiamphenicol and sulfamethoxazole. The composition ratios of antibiotic detection rates were also lower in almost all non-T2DM populations than they were in the T2DM populations, except for chlortetracycline, doxycycline, clarithromycin, sulfadiazine and sulfamethoxazole.

Daily doses of antibiotic exposure were mostly lower in the 95th and 99th percentiles of the non-T2DM population than they were in the T2DM population, except for tetracycline, ofloxacin, sulfamethoxazole and florfenicol (Table 2).

Among 525 middle-aged and older people in Xinjiang, 40 individuals (7.7%) had an HI value ≥ 1 and a health risk. The values of the HQ were mostly higher for the T2DM than they were in the non-T2DM population, except for individual antibiotics such as tetracycline, norfloxacin, ofloxacin, florfenicol and sulfamethoxazole. Notably, the 99th percentile HQ values of oxytetracycline and norfloxacin were relatively high in the T2DM population, at 26.448 and 62.427, respectively, thus indicating that a high oxytetracycline and norfloxacin intake was associated with a high risk of susceptibility to human health effects (Table 3).

Table 4 shows that, in older adults who were 60–74 years of age, compared with middle-aged adults aged 45–59 years, blood glucose values and total antibiotic exposure concentrations increased with age. This trend was also seen with an increasing frequency of vegetable consumption; participants who ate vegetables daily had higher blood glucose values and total antibiotic exposure concentrations than did those who did not eat vegetables daily. Moreover, occasional pork eaters had higher blood glucose values but lower total antibiotic concentrations than did those who did not eat pork, and occasional alcohol drinkers had higher blood glucose values but lower total antibiotic concentrations than did non-drinkers.

The binary logistic regression results indicate that, after adjustment for covariates in Model A, the HI for microbiological effects, HI for PVAs, HQ for norfloxacin and HQ for ciprofloxacin were associated with a higher risk of T2DM in participants with higher exposure risk (HI > 1 or HQ > 1), and this association persisted after adjustment for more covariates in Models B and C (Table 5). Specifically, the risk of T2DM in the group with a microbiological effect HI > 1 was 2.948 times greater than the risk of T2DM in the group with microbiological effect HI ≤ 1 after adjustment for covariates in Model A, with a 95% *CI* and an OR of 2.948 (1.287–6.756). The confidence intervals were 3.391 (1.417–8.120) and 3.442 (1.423–8.327) after adjustment for covariates in Model B and Model C, respectively. After adjustment for covariates in Model A, the risk of T2DM was 2.928 times higher in those with an HI > 1 for PVAs than it was in the group with an HI ≤ 1 for PVAs, with a confidence interval of 2.928 (1.279–6.706). After adjustment for covariates in Models B and C, the confidence intervals were 3.271 (1.371–7.802) and 3.348 (1.386–8.083). After adjustment for covariates in Model A, those in the HQ > 1 group for norfloxacin had 10.075 times the T2DM risk of those in the HQ ≤ 1 group for norfloxacin, with a credible interval of 10.075 (1.612–62.952), and they still had a higher risk after adjustment for covariates in Models B and C, with credible intervals of 11.243 (1.728–73.142) and 10.511 (1.571–70.344). The risk of T2DM was 4.789 times greater in the ciprofloxacin HQ > 1 group than it was in the ciprofloxacin HQ ≤ 1 group after adjustment for covariates in Model A, with a confidence interval of 4.789 (1.336–17.175), and also after adjustment for covariates in Model B and Model C, with confidence intervals of 5.241 (1.377–19.946) and 6.565 (1.676–25.715).

## 4. Discussion

In this study, we measured the concentrations of 18 common antibiotics in urine and then calculated the DED, HQ and HI, thus indicating the relationship between T2DM and antibiotic risk in middle-aged and older people. The HI for microbiological effects >1, HI for PVA use >1, HQ for norfloxacin >1 and HQ for ciprofloxacin >1 in middle-aged and older people were positively associated with T2DM. These associations persisted after adjustment for several confounding factors known to be associated with T2DM. To our knowledge, this study is the first to use urinary antibiotic biomonitoring to explore different sources of antibiotic exposure, and to assess the health risks and consequent effects on T2DM.

**Figure 1:**
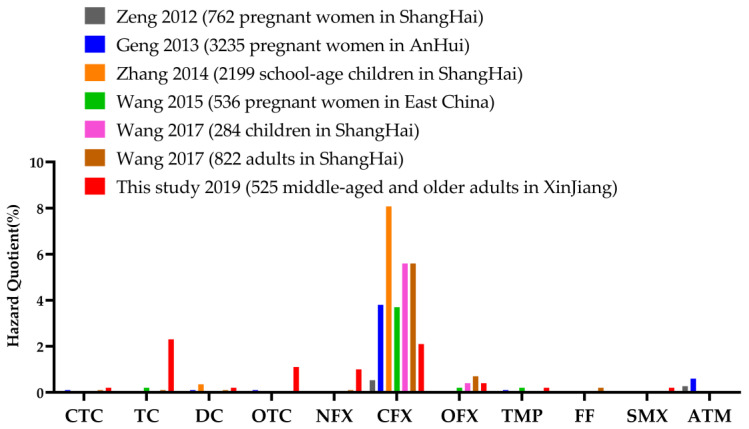
Comparison of hazard quotients of 11 antibiotics in urine between this study and six previous studies conducted by Zeng et al. [28], Geng et al. [29], Zhang et al. [30], Wang et al. [20], Wang et al. [31] and Wang et al. [6]. (CTC: Chlortetracycline; TC: Tetracycline; DC: Doxycycline; OTC: Oxytetracycline; NFX: Norfloxacin; CFX: Ciprofloxacin; OFX: Ofloxacin; TMP: Trimethoprim; FF: Florfenicol; SMX: Sulfamethoxazole: ATM: Azithromycin).

Studies based on the biomonitoring of urinary antibiotic concentrations are lacking. Several studies have been performed in China and Korea [23,24,25,26,27]. Few studies have evaluated the HQ and HI of urinary antibiotics, all of which have been in China (Figure 1) [6,20,28,29,30,31]. Figure 1 shows the comparison of HQs in this study and six previous studies. The HQ levels in Xinjiang middle-aged and older adults were generally exceeded for 11 antibiotics, thus indicating a health risk, particularly for tetracycline (2.3%) and ciprofloxacin (2.1%). Throughout the western and eastern regions of China, the HI for ciprofloxacin was above the human health level threshold in all six studies. The detection rate of a ciprofloxacin HQ > 1 in middle-aged and older adults in Xinjiang was lower than that in several other regions (2.1%). Shanghai school-age children had the highest (8.07%), and the levels of ciprofloxacin HQ > 1 were comparable in Shanghai children and adults, both at 5.6%, and were higher than those in pregnant women in Eastern China (3.7%) and Anhui (3.8%). However, the levels of human antibiotic health risk in each of these regions varied, likely because of differences in locations, lifestyle habits and dietary and drinking habits among regions [32,33,34].

All 17 antibiotics have short half-lives of <15 h, except for azithromycin, which has a half-life of approximately 40 h [35]. Previous studies have hypothesized that exposure levels to antibiotics based on a single sampling test may represent previous long-term exposure levels, because antibiotic exposure depends on antibiotic use, and human exposure to antibiotics in daily life occurs primarily through contaminated food and drinking water (HAs, VAs and PVAs). Therefore, the concentration of urinary antibiotics in a single sample should reflect long-term antibiotic exposure to some extent, but not the intensity of antibiotic use or health risk [23]. This hypothesis is supported by our findings, which suggest that people with T2DM have higher biomonitoring urinary antibiotic concentrations, detection rates, daily exposure doses and HQ levels than do people without T2DM, and that people with diabetes have relatively higher HQ values for oxytetracycline and norfloxacin and have a higher health risk. Because the gut microbiome differed between the T2DM population and non-T2DM population, and the gut microbiome components are closely associated with glucose metabolism (e.g., insulin secretion, insulin sensitivity, etc.), the levels of antibiotic exposure should differ between these groups [36,37,38,39,40]. Because oxytetracycline and norfloxacin are common therapeutic agents in diabetes treatment, high and prolonged doses of oxytetracycline or norfloxacin may cause health risks in patients with diabetes [41].

A growing body of evidence from experimental studies suggests a causal relationship between the gut microbiota and diabetes [42]. Age factors, particularly aging, have been shown to be predisposing factors for abnormal glucose metabolism and abnormal regulation [43]. The diversity of the gut microbiota is diminished and is less stable in adults who are older than middle-age [44]. Antibiotics have multiple effects on host physiology, particularly on glucose homeostasis, by disrupting the intestinal microbiota. These effects are consistent with our findings, indicating an increase in antibiotics and glucose with age in people who are older than middle-age. In contrast, different antibiotic exposures arising from different diets, and the recognition of short-chain fatty acids and secondary bile acids by enteroendocrine cells, the vagus nerve and enteric neurons, among other aspects, result in different effects on glucose homeostasis [45,46]. Eating vegetables and pork and drinking alcohol with different frequencies in our study were associated with different trends in blood glucose levels and antibiotic concentrations. In this study, we observed higher blood glucose values in people who ate pork occasionally than those in people who did not eat pork; in people who ate vegetables daily than those in people who did not eat vegetables daily; and in people who drank alcohol occasionally than those in people who did not drink alcohol. Because pork consumption is a known risk factor for developing T2DM [47,48], and pigs are raised with antibiotics that promote growth and sterilization [49], people with T2DM are more likely to be tested for antibiotic exposure and to experience health risks from antibiotic exposure. A low intake of vegetables increases the risk of developing T2DM [50]. Vegetables are grown with added antibiotics [51], thus potentially increasing antibiotic exposure and risk in people with T2DM. Similarly, alcohol consumption is strongly associated with the development of T2DM, and excessive alcohol consumption can even trigger the risk of cancer in people with T2DM [52,53]. In contrast, alcohol production is prone to contamination with phytotoxins and low pH contaminants, such as antibiotics [54]. Therefore, antibiotics are often detected in patients with T2DM.

Although clinical studies have reported a relationship between antibiotics and T2DM [8,9,12,13,14,55,56], no studies have assessed the association between antibiotic exposure in daily life and the risk of T2DM. Previous findings from a clinical perspective are consistent with the results of this study, in that people who used antibiotics for longer periods of time had a higher risk of developing T2DM than did people who did not use antibiotics; moreover, those who used multiple antibiotics had a higher risk of developing T2DM than did those who used a single antibiotic. A significant dose-dependent relationship has been reported between antibiotic exposure and the incidence of T2DM [8,9,13]. Antibiotic exposure significantly increases the risk of T2DM in people above 50 years of age [14]. Moreover, fluoroquinolones are associated with an increased risk of diabetes [8]. However, results have varied among studies; for example, several studies have reported that single antibiotic use is not associated with the risk of developing T2DM [8,9]. Participants receiving more antibiotics had no increased risk of diabetes [57]. The reason for the discrepancy in the results may be due to differences in the doses and frequencies of medication and the variety of medications used for T2DM [58,59]. HI > 1 for microbiological effects in older people, HI > 1 for preferred veterinary use, HQ > 1 for norfloxacin (a PVA) and HQ > 1 for ciprofloxacin (a PVA) were significantly associated with T2DM and were risk factors for T2DM. These findings suggest that the cumulative health risk triggered by long-term exposure to low doses of antibiotics (contaminated food or drinking water) in daily life can affect T2DM development in middle-aged and older people, and that PVAs should be used in a regulated manner.

This study performed biomonitoring to assess the health risks of middle-aged and older people in Xinjiang. However, it has several limitations. First, this study was a cross-sectional study and thus could not demonstrate a causal relationship between antibiotics and T2DM. Antibiotic exposure might potentially be a proxy for some conditions associated with T2DM. Participants with T2DM might have had different levels of exposure to antibiotics in food or drinking water, and thus different levels of assessed health risks, depending on their dietary structure and intake. Second, because people are intermittently exposed to antibiotics in their daily lives, urinary concentrations in people may vary throughout the day. Antibiotics have short half-lives, and we collected morning urine from participants, thus potentially underestimating the exposure and the health risk assessment after multiple exposures to antibiotics. Single urine samples may not reflect the levels of long-term antibiotic exposure, thus potentially weakening the association between the risk of antibiotic exposure and T2DM in middle-aged and older adults.

## 5. Conclusions

We used a biomonitoring approach to assess antibiotic exposure and thus the risk of antibiotic exposure, and we found that exposure to antibiotics in daily life (mainly from food or drinking water contaminated with antibiotics), particularly with an HI > 1 for microbiological effects, HI > 1 for PVAs, HQ > 1 for norfloxacin and HQ > 1 for ciprofloxacin, may be risk factors associated with T2DM in middle-aged and older people. Because cross-sectional studies and primary urine sampling reflect long-term antibiotic exposure, more prospective studies and experimental studies are needed to further explore these associations and their underlying mechanisms.

## Figures and Tables

**Table 1:** Frequency and concentration distribution of antibiotics detected in the urine of middle-aged and older people with and without type 2 diabetes in Xinjiang (*n* = 525).

Antibiotic	Usage	All (*n* = 525) *n* (%) ^a^	Non-T2DM (*n* = 461)				T2DM (*n* = 64)			
			*n* (%) ^a^	Percentiles		Maximum	*n* (%) ^a^	Percentiles		Maximum
				95th	99th			95th	99th	
All antibiotics ^b^		268 (51.0)	228 (49.5)	78.09	20,991.06	44,588.26	40 (62.5)	2363.48	24,282.26	24,282.26
HAs		54 (10.3)	43 (9.3)	0.87	77.06	4615.54	11 (17.2)	41.30	2989.35	2989.35
VAs		60 (11.4)	48 (10.4)	2.02	148.17	36,112.85	12 (18.8)	4.48	2090.43	2090.43
PVAs		224 (42.7)	190 (41.2)	44.97	7343.72	44,418.14	34 (53.1)	272.99	24,282.26	24,282.26
Tetracyclines ^c^		79 (15.0)	64 (13.9)	13.35	20,991.06	44,565.07	15 (23.4)	42.71	2362.84	2362.84
Chlortetracycline	VA	4 (0.8)	4 (0.9)	—	—	119.02	0 (0)	—	—	—
Tetracycline	PVA	60 (11.4)	50 (10.8)	7.41	626.39	44,416.90	10 (15.6)	12.48	272.42	272.42
Doxycycline	PVA	3 (0.6)	3 (0.7)	—	—	11,315.50	0 (0)	—	—	—
Oxytetracycline	VA	38 (7.2)	30 (6.5)	1.43	75.75	36,112.85	8 (12.5)	4.48	2090.43	2090.43
Fluoroquinolones ^c^		170 (32.4)	142 (30.8)	7.09	726.97	2876.96	28 (43.8)	67.69	24,282.26	24,282.26
Enrofloxacin	VA	11 (2.1)	7 (1.5)	—	0.21	9.40	4 (6.3)	0.20	0.96	0.96
Norfloxacin	PVA	66 (12.6)	55 (11.9)	1.28	18.14	1012.23	11 (17.2)	1.11	24,282.26	24,282.26
Ciprofloxacin	PVA	35 (6.7)	29 (6.3)	0.57	6.24	928.11	6 (9.4)	3.86	8.66	8.66
Ofloxacin	PVA	105 (20.0)	92 (20.0)	1.63	39.86	2876.96	13 (20.3)	1.08	66.89	66.89
Macrolides ^c^		29 (5.5)	24 (5.2)	0.87	77.06	4615.54	5 (7.8)	41.30	2989.35	2989.35
Azithromycin	HA	15 (2.9)	13 (2.8)	—	5.11	479.12	2 (3.1)	—	248.70	248.70
Clarithromycin	HA	3 (0.6)	3 (0.7)	—	—	4615.54	0 (0)	—	—	—
Roxithromycin	HA	15 (2.9)	12 (2.6)	—	23.10	132.94	3 (4.7)	—	2989.35	2989.35
Sulfonamides ^c^		47 (9.0)	40 (8.7)	0.25	1.81	7232.59	7 (10.9)	0.21	2.74	2.74
Sulfamethazine	PVA	16 (3.0)	13 (2.8)	—	0.28	11.66	3 (4.7)	—	0.28	0.28
Sulfadiazine	PVA	1 (0.2)	1 (0.2)	—	—	10.51	0 (0)	—	—	—
Sulfamethoxazole	PVA	9 (1.7)	9 (2.0)	—	0.69	5546.27	0 (0)	—	—	—
Trimethoprim	PVA	33 (6.3)	29 (6.3)	0.12	1.12	1686.32	4 (6.3)	0.06	2.74	2.74
Phenicols ^c^		46 (8.8)	37 (8.0)	0.05	0.63	30.93	9 (14.1)	0.24	1.04	1.04
Chloramphenicol	HA	30 (5.7)	23 (5.0)	—	0.12	9.98	7 (10.9)	0.05	0.78	0.78
Florfenicol	VA	18 (3.4)	15 (3.3)	—	0.43	9.86	3 (4.7)	—	0.43	0.43
Thiamphenicol	PVA	3 (0.6)	2 (0.4)	—	—	11.10	1 (1.6)	—	0.08	0.08

HA: human antibiotic; VA: veterinary antibiotic; PVA: preferred veterinary antibiotic. ^a^ Positive detection (detection frequency, %). ^b^ Positive detection of one or more studied antibiotics. ^c^ Positive detection of one or more antibiotics in the corresponding category. —Urinary concentration of antibiotics is below the limit of detection.

**Table 2:** Estimated daily intake of antibiotics (μg/kg/day), on the basis of urinary concentrations of antibiotics in middle-aged and older people in Xinjiang (*n* = 525).

	Non-T2DM (*n* = 461)			T2DM (*n* = 64)		
	Percentiles		Maximum	Percentiles		Maximum
Antibiotic	95th	99th		95th	99th	
Tetracyclines ^a^	0.44	569.61	1619.25	1.27	91.25	91.25
Chlortetracycline	0.00	0.00	5.44	0.00	0.00	0.00
Tetracycline	0.32	25.64	1612.89	0.45	11.91	11.91
Doxycycline	0.00	0.00	336.85	0.00	0.00	0.00
Oxytetracycline	0.06	3.11	1443.79	0.16	79.34	79.34
Fluoroquinolones ^a^	0.22	37.14	57.11	2.13	873.98	873.98
Enrofloxacin	0.00	0.03	0.89	0.03	0.11	0.11
Norfloxacin	0.05	0.76	45.57	0.04	873.98	873.98
Ciprofloxacin	0.03	0.24	51.21	0.17	0.37	0.37
Ofloxacin	0.04	0.96	57.11	0.02	2.09	2.09
Macrolides ^a^	0.04	4.59	383.10	1.01	108.08	108.08
Azithromycin	0.00	1.57	167.41	0.00	108.08	108.08
Clarithromycin	0.00	0.00	383.10	0.00	0.00	0.00
Roxithromycin	0.00	0.83	3.85	0.00	94.88	94.88
Sulfonamides ^a^	0.01	0.12	610.71	0.01	0.10	0.10
Sulfamethazine	0.00	0.01	0.35	0.00	0.01	0.01
Sulfadiazine	0.00	0.00	0.40	0.00	0.00	0.00
Sulfamethoxazole	0.00	0.07	536.56	0.00	0.00	0.00
Trimethoprim	0.00	0.05	74.15	0.00	0.10	0.10
Phenicols ^a^	0.00	0.03	1.02	0.01	0.03	0.03
Chloramphenicol	0.00	0.00	0.21	0.00	0.02	0.02
Florfenicol	0.00	0.03	0.41	0.00	0.02	0.02
Thiamphenicol	0.00	0.00	0.40	0.00	0.00	0.00
All antibiotics ^b^	3.82	615.82	1619.98	95.29	873.98	873.98

^a^ Sum of estimated daily intakes of antibiotics in the corresponding category. ^b^ Sum of estimated daily intake of all antibiotics.

**Table 3:** Hazard quotient (HQ) of veterinary antibiotics and human/veterinary antibiotics among middle-aged and older people in Xinjiang (*n* = 525).

Antibiotics	Effect End Point	ADI (μg/kg/day)	Overall *N* (%)	Non-T2DM (*n* = 461)			T2DM (*n* = 64)		
				Percentiles		Maximum	Percentiles		Maximum
				95th	99th		95th	99th	
Hazard index ^a^									
Chlortetracycline	Microbiological	3	1 (0.2)	0.000	0.000	1.813	0.000	0.000	0.000
Tetracycline	Microbiological	3	12 (2.3)	0.107	8.547	537.630	0.150	3.970	3.970
Doxycycline	Microbiological	3	1 (0.2)	0.000	0.000	112.284	0.000	0.000	0.000
Oxytetracycline	Microbiological	3	6 (1.1)	0.019	1.038	481.264	0.052	26.448	26.448
Enrofloxacin	Microbiological	6.2	0 (0)	0.000	0.005	0.143	0.004	0.017	0.017
Norfloxacin	Microbiological	14	5 (1.0)	0.004	0.054	3.255	0.003	62.427	62.427
Ciprofloxacin	Microbiological	0.15	11 (2.1)	0.202	1.589	341.393	1.129	2.464	2.464
Ofloxacin	Microbiological	3.2	2 (0.4)	0.014	0.299	17.847	0.008	0.653	0.653
Trimethoprim	Microbiological	4.2	1 (0.2)	0.001	0.012	17.656	0.001	0.025	0.025
Florfenicol	Microbiological	3	0 (0)	0.000	0.010	0.137	0.000	0.007	0.007
Thiamphenicol	Microbiological	2.5	0 (0)	0.000	0.000	0.159	0.000	0.002	0.002
Hazard index ^b^									
Sulfamethazine	Toxicological	1.6	0 (0)	0.000	0.006	0.220	0.000	0.008	0.008
Sulfadiazine	Toxicological	20	0 (0)	0.000	0.000	0.020	0.000	0.000	0.000
Sulfamethoxazole	Toxicological	130	1 (0.2)	0.000	0.001	4.127	0.000	0.000	0.000

^a^ Sum of HQ based on microbiological effect. ^b^ Sum of HQ based on toxicological effect; N: value of HQ ≥ 1.

**Table 4:** Blood glucose and all antibiotics in relation to demographic variables.

Variable	Blood Glucose (mmol/L) ^a^	*p*-Value ^d^	All Antibiotics (ng/mL) ^c^	*p*-Value ^b^
Gender		0.698		0.090
Male (*n* = 264)	5.90 ± 1.68		0 (1.78)	
Female (*n* = 261)	5.84 ± 1.79		0.11 (2.45)	
Age		0.373		0.041
45–59 (*n* = 432)	5.73 ± 1.43		0 (1.65)	
60–74 (*n* = 227)	6.15 ± 2.18		0.17 (2.27)	
Education		0.457		0.101
≤Primary (*n* = 383)	5.89 ± 1.86		0.08 (1.78)	
Secondary (*n* = 112)	5.92 ± 1.46		0 (1.82)	
≥High school (*n* = 30)	5.49 ± 0.48		0 (0.36)	
Income level		0.022		0.821
<10,000 RMB/year (*n* = 76)	6.27 ± 2.40		0.16 (1.63)	
10,000–35,000 RMB/year (*n* = 332)	5.89 ± 1.71		0.03 (1.69)	
≥35,000 RMB/year (*n* = 116)	5.57 ± 1.14		0 (2.37)	
Physical activity		0.824		0.267
Never (*n* = 427)	5.87 ± 1.78		0.04 (2.09)	
Occasionally (*n* = 98)	5.90 ± 1.52		0 (0.82)	
Pork		0.101		0.094
Do not eat (*n* = 444)	5.82 ± 1.73		0.05 (2.03)	
Occasionally eat (*n* = 78)	6.17 ± 1.77		0 (0.77)	
Beef		0.974		0.478
Do not eat (*n* = 155)	5.88 ± 1.63		0.07 (1.69)	
Occasionally eat (*n* = 281)	5.86 ± 1.88		0 (1.45)	
Eat every day (*n* = 87)	5.91 ± 1.41		0.17 (2.27)	
Mutton		0.057		0.138
Do not eat (*n* = 128)	6.19 ± 2.30		0.03 (1.72)	
Occasionally eat (*n* = 205)	5.82 ± 1.58		0 (0.94)	
Eat every day (*n* = 189)	5.73 ± 1.41		0.08 (3.07)	
Fried food		0.457		0.130
Do not eat (*n* = 328)	5.83 ± 1.64		0.07 (2.03)	
Occasionally eat (*n* = 197)	5.95 ± 1.88		0 (1.41)	
Vegetables		0.274		0.166
Not every day (*n* = 28)	5.52 ± 1.34		0 (0.68)	
Every day (*n* = 497)	5.89 ± 1.75		0.04 (1.78)	
Fruits		0.562		0.997
Not every day (*n* = 384)	5.85 ± 1.68		0.04 (1.69)	
Every day (*n* = 141)	5.95 ± 1.88		0.02 (2.70)	
Smoking		0.539		0.059
Never (*n* = 397)	5.92 ± 1.84		0.10 (2.05)	
Occasionally (*n* = 16)	5.88 ± 1.26		0.14 (4.36)	
Every day (*n* = 112)	5.71 ± 1.39		0 (0.96)	
Drinking		0.408		0.453
Never (*n* = 440)	5.85 ± 1.76		0.04 (1.75)	
Occasionally (*n* = 85)	6.02 ± 1.58		0 (1.39)	

^a^ Mean ± standard deviation. ^b^ Rank test. ^c^ Median (interquartile range). ^d^ *t*-test or ANOVA.

**Table 5:** Associations of urinary antibiotic risk index, risk quotient and diabetes risk in middle-aged and older people, on the basis of binary logistic regression.

	Model A		Model B		Model C	
Variables	Tertile 1	Tertile 2	Tertile 1	Tertile 2	Tertile 1	Tertile 2
**Group based on hazard index**						
HI for microbiological effects	Ref.	2.948 (1.287–6.756) *	Ref.	3.391 (1.417–8.120) *	Ref.	3.442 (1.423–8.327) *
HI for toxicological effects	Ref.	—	Ref.	—	Ref.	—
HI for veterinary antibiotics	Ref.	0.962 (0.109–8.472)	Ref.	1.004 (0.105–9.607)	Ref.	0.952 (0.098–9.243)
HI for preferred veterinary antibiotics	Ref.	2.928 (1.279–6.706) *	Ref.	3.271 (1.371–7.802) *	Ref.	3.348 (1.386–8.083) *
**Group based on hazard quotient**						
HQ for tetracycline	Ref.	1.164 (0.245–5.534)	Ref.	1.195 (0.241–5.926)	Ref.	1.090 (0.218–5.456)
HQ for oxytetracycline	Ref.	1.195 (0.129–11.052)	Ref.	1.398 (0.136–14.372)	Ref.	1.238 (0.118–12.994)
HQ for norfloxacin	Ref.	10.075 (1.612–62.952) *	Ref.	11.243 (1.728–73.142) *	Ref.	10.511 (1.571–70.344) *
HQ for ciprofloxacin	Ref.	4.789 (1.336–17.175) *	Ref.	5.241 (1.377–19.946) *	Ref.	6.565 (1.676–25.715) *

* *p*-value < 0.05. Model A adjusted for age, pork and vegetable consumption, and drinking. Model B adjusted for sex, age, education and income level; pork, mutton, fried food and vegetable consumption; and smoking and drinking. Model C adjusted for sex, age, education, income level and physical activity; pork, beef, mutton, fried food, vegetable and fruit consumption; and smoking and drinking. —: Odds ratios are not provided because the proportion of participants with diabetes with a toxicological effect HQ ≥ 1 was too low.

## Data Availability

Research data will be submitted as required.
